# Rebuild doctor–patient trust in medical service delivery in China

**DOI:** 10.1038/s41598-020-78921-y

**Published:** 2020-12-15

**Authors:** Liang Du, Jia Xu, Xu Chen, Xuexue Zhu, Yu Zhang, Ruiheng Wu, Haoqiang Ji, Ling Zhou

**Affiliations:** School of Public Health, Dalian Medical University, 9 Western Section, Lvshun South Street, Lvshunkou District, Dalian, 116044 Liaoning People’s Republic of China

**Keywords:** Health services, Medical ethics, Patient education, Public health

## Abstract

Doctor–patient trust is not strong in China, but studies examining this factor remain insufficient. The present study aimed to explore the effect of doctor–patient communication, medical service quality, and service satisfaction on patient trust in doctors. Five hundred sixty-four patients with tuberculosis participated in this cross-sectional study in Dalian, China. They completed questionnaires assessing socio-demographic characteristics, doctor–patient communication, medical service quality, service satisfaction and patient trust in medical staff. A structural equation model was applied to examine the hypotheses, and all the study hypotheses were supported: (1) doctor–patient communication, medical service quality and service satisfaction were positively associated with building doctor–patient trust; (2) service quality positively mediated the relationship between doctor–patient communication and trust; (3) medical service satisfaction positively mediated the relationship between doctor–patient communication and trust; (4) medical service satisfaction positively mediated the relationship between medical service quality and doctor–patient trust; and (5) medical service quality and service satisfaction were the positively sequential mediators between communication and doctor–patient trust. Based on these findings, improvements in doctor–patient communication, medical service quality, and service satisfaction are the important issues contributing to the rebuilding of doctor–patient trust in medical service delivery.

## Introduction

A good doctor–patient relationship is the fundamental factor ensuring a normal medical process. Doctor–patient trust is the global attribute of treatment relationships, and it usually encompasses subsidiary features, such as communication, medical service quality and satisfaction^1^. Over time, a gradual deterioration of doctor–patient trust appears to be an inevitable trend for the healthcare system worldwide. A survey study conducted in the USA indicated that 82.2% of patients with chronic diseases trust their doctors^2^. Another study in European regions suggested that the Italian-speaking patients exhibited high trust in doctors, but less than half of the French-speaking patients had trust in doctors^3^. A literature review reported an insufficient level of doctor–patient trust in East Asia, including Mainland China, Hong Kong, Taiwan, South Korea and Japan; patients in China reported suboptimal trust in doctors because of less doctor–patient communication and few shared decision-making processes^4^.

In the past decade, the relationship between the doctor and patient has become increasingly worse in China^5,6^. Frequent incidents of patients performing offending and adverse events against health care workers in the form of verbal and physical abuse, injury, and even murder^7–9^, which is not beneficial to the development of medical service in China^10,11^. The root cause of doctor–patient conflicts is the issue of trust between doctors and patients, particularly patients’ trust towards medical staff^11^. If patients express less trust in doctors, on the one hand, patients will doubt the doctors’ treatment regimen and will not participate in treatment with positivity regarding the outcome, which may lead to non-ideal treatment effects and arouse patients' negative feelings about the doctors’ professional authority and their consideration of doctors as retailers selling medical services^12^. On the other hand, subsequently, doctors observe a substantial decrease in patients’ treatment expectations, which increases the dissatisfaction of doctors with patients, thus leading to the occurrence of adverse events between doctors and patients^13,14^. Patient trust in medical staff has been defined as the belief of patients that doctors have the skills necessary for diagnosis and treatment and that they will first consider the patients’ interests, thereby allowing patients to accept the medical services and be reassured^15^. Doctor–patient trust plays an important role in the medical process, and insufficient trust might be associated with an adverse medical service experience^16^, lower treatment adherence of patients^17^, and even treatment failure^18^. Therefore, studies aiming to explore the mechanism underlying the effect of doctor–patient trust are necessary and urgently needed.

Although doctor–patient trust is not optimistic in China, few studies have been conducted on doctor–patient trust. Various intricate factors may be associated with doctor–patient trust, including socioeconomic characteristics, medical personnel, medical service institutions, and social networking services^6,19–21^. However, knowledge of whether the medical service delivery process is related to doctor–patient trust is limited. A recent quantitative study using dyadic analysis found that a higher quality of communication positively predicted doctor–patient trust^22^. Additionally, the medical service quality, which was mainly measured based on the patient’s perception and feelings, was identified as another important factor associated with doctor–patient trust^23^. Besides, doctor–patient trust significantly predicted patients' satisfaction with medical services in a previous study^24^, but few studies have yet explored the effect of service satisfaction on doctor–patient trust.

When receiving medical services, patients are unable to perceive the quality of and satisfaction with medical services if communication between doctors and patients is lacking. Ensuring the quality of medical services is an important part of operating a hospital, which is also beneficial to improving the health of a sub-healthy population^25^. The assessment of the medical service quality may be difficult and complex in practice, but doctors’ attitudes, medical behaviours, and professional skills are shared among studies^26,27^. Medical service quality is described as conforming to three dimensions, outcome, environmental quality, and interaction, among which the interaction quality exhibited the greatest effect on overall service quality. They also concluded that service-related attitudes, behaviours, and expertise are components of interaction quality, namely, functional quality. Based on cognitive psychology theory. The concept of customer-perceived service quality depends on the comparison between service expectation and perceived actual service performance, in which only when perceived actual service performance was greater than service expectation would customers perceive a high level of service quality. With the peculiarity of medical services, doctor–patient communication is associated with what medical services the doctors would provide, which may eventually influence the medical service quality that patients receive^28,29^.

Patient-perceived service satisfaction might be considered as a prospective outcome of healthcare, which incorporates interpersonal relationships, specific components of medical technology, and the outcomes of care^26^. Measurements of satisfaction with medical services are a complicated issue. Although the measurement of patient satisfaction has been studied for years, a consensus on its definition and the best methodology to measure it has not been achieved. Dissatisfactory medical service is a symptom of one flawed system that victimizes both patients and doctors, and directly leads to violence in the medical process^7,11^. According to stimulus response theory, when an individual receives a stimulus from the outside world, he/she may form new behaviours or attitudes in the future if the contents of the stimulus are accepted^30^. Previous studies have confirmed that high-quality doctor–patient communication contributes to patients' satisfaction, which then may tend to establish the patients' trust in medical staff^11,31,32^.

Social exchange theory (SET) is a value expectancy theory that involves an assessment between individual expectations of the outcomes of an action, as well as their subjective values for those outcomes^33^. If the benefits of the outcome are more rewarding than the cost of the activity, this relationship will be valued and the individual will be more likely to engage in the collaborative behaviour again in the future. In medical service delivery, patients require better treatment and quality of service, which requires that patients trust in doctors; doctors tend to pursue patients’ satisfaction and trust, both of which require effective communication with each other. SET suggests that both doctors and patients are motivated to improve communication in medical service delivery if they expect a positive outcome, indicating that doctors would provide the best service possible to meet the patients’ needs^34^. With high medical service quality supplied by doctors, the patients may have a greater sense of satisfaction and trust towards their doctors.

**Figure 1:**
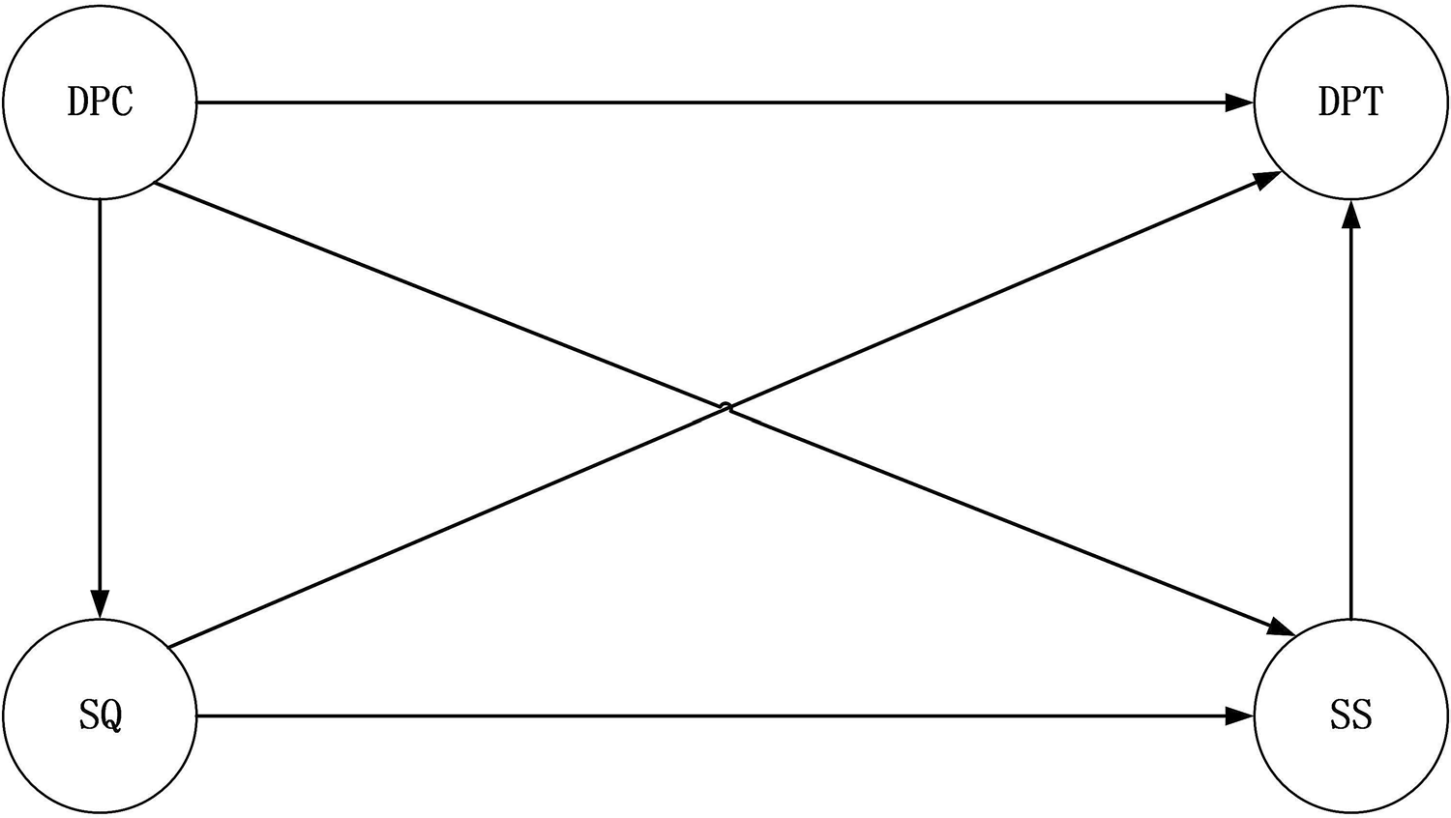
The research model based on the hypotheses. *DPC* doctor–patient communication, *SQ* service quality, *SS* service satisfaction, *DPT* doctor–patient trust.

China is now the country with the second highest tuberculosis (TB) burden in the world, and TB is also the crucial infectious disease threatening the health of Chinese people^35,36^. In the anti-TB treatment process, the problem of distrust between doctors and patients occurs frequently, mainly because of the factors listed below. (1) Although China has been implementing a policy of free treatment for TB, patients still face a serious financial burden^37^. (2) The privacy of TB patients is a prominent concern, and patients may fear that doctors reveal their tuberculosis status to others, which will lead to social discrimination, alienation, and stigma from public^38,39^. (3) During the continuation phase of anti-TB treatment, some patients experience unresolved TB symptoms or adverse drug reactions, although they are being treated with their regular medication^40^. Suboptimal patient trust in doctors gives rise to doctor–patient conflict and prejudice, and patients’ poor treatment adherence, which may further lead to the aggravation of their illness, treatment interruption or failure, relapse, infection of others, and obstruction of the progress of TB treatment and control in China. Based on these findings and using patients with TB as study sample, the purpose of present study was to test the hypotheses (see Fig. 1) that (1) doctor–patient communication, medical service quality and service satisfaction directly benefited building doctor–patient trust (H1); (2) service quality mediated the relationship between doctor–patient communication and doctor–patient trust (H2); (3) medical service satisfaction mediated the relationship between doctor–patient communication and doctor–patient trust (H3); (4) medical service satisfaction mediated the relationship between medical service quality and doctor–patient trust (H4); and (5) medical service quality and service satisfaction were the sequential mediators from doctor–patient communication to doctor–patient trust (H5). According to these research hypotheses, we attempted to describe methods to rebuild doctor–patient trust in medical service delivery.

## Methods

### Study design and setting

This multi-centre survey was conducted between June 20, 2019, and August 31, 2019, and four medical institutions in Dalian, Liaoning Province, in northeast China were involved^41^. The four TB medical institutions, which served different types of patients, were chosen according to their institution level and location. The first was a tertiary hospital that mainly serves patients across the whole city, particularly critically ill and urban patients. The second was a county hospital that serves both rural and urban patients with TB. The other two medical institutions were tuberculosis dispensaries, which only serve local patients with a milder disease.

### Participants

Outpatients with pulmonary TB who had been receiving treatment for > 2 months were eligible participants. We excluded patients aged < 15 years and patients who were unable to complete the questionnaire due to a mental or psychological illness. All participants were told that their privacy would be protected and they were free to withdraw from the study at any time. The participants signed an informed consent form prior to participation in our study. A parent or legal guardian provided informed consent for any patient under the age of 18. Detail description can be found in our another study^41^.

### Ethics procedure

The study protocol was reviewed and approved by the ethics committee of Dalian Medical University, Liaoning province, China. All methods in our study were carried out in accordance with relevant guidelines and regulations (Declaration of Helsinki).

### Measurement

Socio-demographic characteristics of the participants were collected, including gender, age, marriage, immigration status, residence, education, monthly income and hospital type. Additionally, doctor–patient communication, medical service quality, service satisfaction and doctor–patient trust were measured in this study.

#### Doctor–patient communication

The communication between doctor and patients was measured with a 4-item scale, which was a component of the Consumer Assessment of Healthcare Providers and Systems (CAHPS) that involved doctor–patient communication, access to needed medical service, timely healthcare, and other factors^42^. Response options for each item ranged from 1 = never to 5 = always. Higher scores indicate low-level communication. The reliability coefficient of Cronbach’s α = 0.89 was for the original CAHPS scale^43^ and α = 0.85 in this study.

#### Medical service quality

Based on a literature review and expert consultation, we compiled three items to measure service quality. We asked the patients about their experience from three aspects: (1) the medical workers did their best to meet your healthcare and nursing needs; (2) the skills and expertise of the medical workers were very good; (3) the attitudes and behaviours of the medical workers were very careful. Participants responded to the 3 items on a 5-point Likert-type scale (1 = strongly disagree to 5 = strongly agree). The reliability coefficient of Cronbach’s α for the 3-item scale was 0.65.

#### Service satisfaction

Participants in this study are outpatients with TB who have frequent hospital visits, and thus we considered that the main components of perceived service satisfaction were doctors’ explanations of patient health conditions, precautions related to the application of anti-TB drugs, and lifestyle and health guidance, such as diet and physical exercise. Based on a literature review and expert consultation, we compiled three items to measure service satisfaction: (1) how satisfied are you with the doctor's explanation of your disease; (2) how satisfied are you with the doctor's explanation of the drug used and its side effects; and (3) how satisfied are you with the doctor's instructions regarding your life and health. Participants responded to the 3 items on a 5-point Likert-type scale (1 = strongly dissatisfied to 5 = strongly satisfied). The reliability coefficient of Cronbach’s α for the 3-item scale was 0.82.

#### Doctor–patient trust

The self-reported trust of the patient in the doctor was measured using the sub-scale of the Trust in Physician scale, which consisted of four items: (1) you completely trust your doctor’s decisions about which treatments are best for you; (2) your doctor only thinks about what is best for you; (3) you have no worries about putting your life in your doctor’s hands; and (4) overall, you have complete trust in your doctor^44,45^. Trust was divided into various dimensions, and this 4-item subscale measured the patients’ global trust in doctors. Participants responded to the four items on a 5-point Likert-type scale (1 = strongly disagree to 5 = strongly agree). The reliability coefficient of Cronbach’s α = 0.93 for the Trust in Physician scale^45^, and α = 0.85 for the 4-item subscale in this study.

### Statistical analysis

A sample of 593 records containing the questionnaire data was collected, and we used EpiData software version 3.1 (The EpiData Association, Odense, Denmark) to establish a database to enter the questionnaire data. We deleted records containing > 20% missing values, which led to the inclusion of 564 eligible records in the final sample. We mainly used frequencies and percentages to describe the study sample.

When considering more than one potential mediating variable and complex relationships in the research model, we used the structural equation model (SEM) to test the hypotheses. SEM finds the potential and important associations to produce a more complete picture of the causal effect mechanism, and it specifically incorporates the measurement error in the research model^46^. Additionally, the confirmatory factor analysis (CFA) was performed to examine the validity and reliability of constructs and was combined with SEM to improve the research model^46^. Factor loading (F.L.), composite reliability (CR), average of variance extracted (AVE) and discriminant validity were reported. Additionally, the comparative fit index (CFI), Tucker–Lewis index (TLI), root mean squared error of approximation (RMSEA), and standardized root mean square residual (SRMR) were calculated to test the fit of the model. The mediation models were constructed using bootstrapping, a nonparametric test that does not rely on assumptions of a normal distribution. Using this method, a significant indirect effect indicated by a 95% confidence interval (CI) not including zero provides evidence for the presence of mediation.

The SPSS 25.0 statistical package (IBM Corporation, Armonk, State of New York) was used to preliminarily analyse data obtained from the survey. According to the principle of the structural equation model, our conceptual model was then tested using Mplus 7.0 statistical analysis software (Muthén & Muthén, Los Angeles, CA, USA), and the multivariable linear regression model was used in our study. Two-sided *P* < 0.05 was considered to indicate statistical significance.

## Results

### Sample description

Among the 564 participants, approximately twice as many male patients (66.31%) as female patients (33.69%) were included. The mean age was 47.41 years and the median was 49 years (interquartile range 31 years). Most of the participants were married (71.10%). Only 93 (16.49%) were migrants, and slightly more rural patients (52.66%) than urban patients (47.34%) were analysed. Middle school education (34.22%) was the most common education level, and the percentages among the other education levels were similar. The number of the participants decreased with the increase in monthly income and only 61 participants (10.82%) reported a monthly income of more than 5000 yuan. The linear regression analysis showed that only hospital type was significantly associated with doctor–patient trust, and the county hospital exerted the greatest effects on this outcome (Table 1).

**Table 1:** Socio-demographic characteristics and the associations with doctor–patient trust.

Variable	Description	N (%)	Est. (*b)*	*P-value*
Gender	Male	374 (66.31%)	Reference	
	Female	190 (33.69%)	0.146	0.530
Age (years)	< 21	26 (4.61%)	Reference	
	21–40	196 (34.75%)	0.112	0.835
	41–60	181 (32.09%)	0.021	0.971
	> 60	161 (28.55%)	0.061	0.920
Marriage	Married	401 (71.10%)	Reference	
	Unmarried/widowed	163 (28.90%)	0.007	0.980
Immigration	Yes	93 (16.49%)	Reference	
	No	471 (83.51%)	− 0.029	0.919
Residence	Urban	267 (47.34%)	Reference	
	Rural	297 (52.66%)	− 0.088	0.755
Education	Primary or below	123 (21.28%)	Reference	
	Middle school	193 (34.22%)	0.208	0.488
	High school	120 (21.28%)	0.293	0.436
	College or above	128 (22.70%)	0.239	0.564
Income (yuan/month)	< 1000	223 (39.54%)	Reference	
	1000–3000	129 (22.87%)	0.347	0.213
	3000–5000	151 (26.77%)	0.366	0.207
	> 5000	61 (10.82%)	0.089	0.823
Hospital type	Tertiary hospital	199 (35.38%)	Reference	
	County hospital	152 (26.95%)	1.605	< 0.001
	TB dispensaries	213 (37.77%)	0.607	0.037

### Reliability and validity of the constructs

According to the factor analysis, all factor loading values for the items were > 0.5, implying that these items measured the constructs well. The lowest value of the CR was 0.669, indicating that the constructs exhibited acceptable composite reliability. Additionally, the values of $\sqrt{\mathrm{AVE}}$s were greater than the values of the *Pearson* correlation coefficients between constructs in the same row or column, which exhibited ideal discriminant validity. In summary, these constructs exhibited good reliability and validity^47^ (Table 2).

**Table 2:** Results of the reliability and validity tests.

Construct	Items	F.L. range	CR	AVE	Discriminant validity			
					DPC	SQ	SS	DPT
DPC	4	0.794–0.811	0.879	0.645	**0.803**			
SQ	3	0.546–0.734	0.669	0.406	0.465	**0.637**		
SS	3	0.670–0.869	0.828	0.620	0.659	0.485	**0.787**	
DPT	4	0.682–0.828	0.853	0.593	0.548	0.616	0.565	**0.770**

The bold values shown on the diagonal are $\sqrt{\mathrm{AVE}}$s, and the values under the bold value represent the *Pearson* correlation coefficients between constructs.

*DPC* doctor–patient communication, *SQ* service quality, *SS* service satisfaction, *DPT* doctor–patient trust, *F.L.* factor loading, *CR* composite reliability, *AVE* average of variance extracted.

### Test of the fitting index of the research model

Using the maximum likelihood method, the research model exhibited an ideal goodness of fit and the fitness indexes all satisfied the practical criteria or threshold values (Chi-square test of the model fit = 210.535; degrees of freedom = 71; Chi-square/DF < 3; CFI = 0.962 > 0.95; TLI = 0.952 > 0.95; RMSEA = 0.059 < 0.08; SRMR = 0.039 < 0.08), indicating that the data fitted our conceptual research model very well^48^ (Table 3).

**Table 3:** Fitting index of the research model.

Index	Criteria	Research model	Support or not
Chi-square	Smaller is better	210.535	Support
DF	Larger is better	71	Support
Chi-square/DF	3 > Chi-square/DF > 1	2.96	Support
CFI	> 0.90	0.962	Support
TLI	> 0.90	0.952	Support
RMSEA	< 0.08	0.059	Support
SRMR	< 0.08	0.039	Support

*DF* degrees of freedom, *CFI* comparative fit index, *TLI* Tucker–Lewis index, *RMSEA* root mean squared error of approximation, *SRMR* standardized root mean square residual.

### Results of the linear regression model

**Figure 2:**
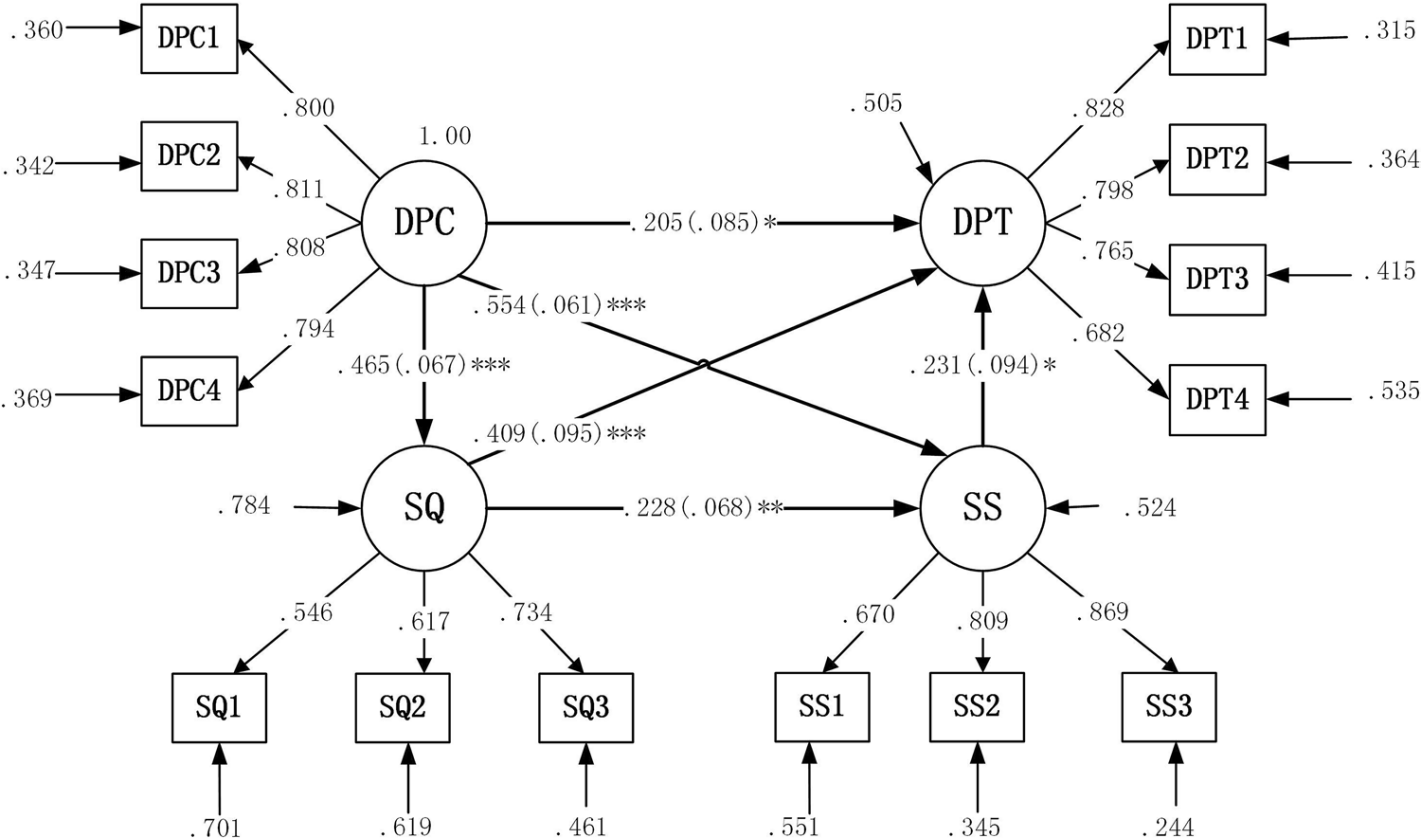
The pathway analysis of the factors influencing doctor–patient trust. The values in the brackets represent the standard error. *P < 0.05, **P < 0.01, and ***P < 0.001. *DPC* doctor–patient communication, *SQ* service quality, *SS* service satisfaction, *DPT* doctor–patient trust. Fitting of model: Chi-square test of the model fit = 210.535; degrees of freedom = 71; RMSEA = 0.059; CFI = 0.962; TLI = 0.952; SRMR = 0.039.

Doctor–patient communication positively predicted the service quality (*b* = 0.465, *P* < 0.001), and the *R-square* value was 0.216. Additionally, satisfaction with the medical service was also significantly and positively interpreted by doctor–patient communication (*b* = 0.554, *P* < 0.001) and service quality (*b* = 0.228, *P* = 0.001), and the *R-square* value was 0.476. Finally, doctor–patient trust was positively interpreted by doctor–patient communication (*b* = 0.205, *P* = 0.016), service quality (*b* = 0.409, *P* < 0.001) and service satisfaction (*b* = 0.231, *P* = 0.014), and the *R-square* value was 0.495. Obviously, the first hypotheses were supported by these results (Table 4 and Fig. 2).

**Table 4:** Research model regression weight and hypothesis.

DV	IV	Est. (*b*)	S.E.	Est./S.E.	*P-value*	Bootstrap 1000 times 95% CI (bias-corrected)		*R-square*
						Lower	Upper	
SQ	DPC	0.465	0.067	6.933	< 0.001	0.330	0.586	0.216
SS	DPC	0.554	0.061	9.084	< 0.001	0.424	0.646	0.476
	SQ	0.228	0.068	3.344	0.001	0.094	0.363	
DPT	DPC	0.205	0.085	2.419	0.016	0.050	0.379	0.495
	SQ	0.409	0.095	4.328	< 0.001	0.248	0.618	
	SS	0.231	0.094	2.455	0.014	0.078	0.464	

*DPC* doctor–patient communication, *SQ* service quality, *SS* service satisfaction, *DPT* doctor–patient trust, *S.E.* standard error, *DV* dependent variable, *IV* independent variable, *CI* confidence interval.

### Total and indirect effects on doctor–patient trust

Using the bootstrapping method, 95% bias-corrected confidence intervals (CIs) without “zero” indicate significant mediating and total effects^49^. The total effect of service quality on doctor–patient trust was 0.462 (95% CI (0.303, 0.656)). Medical service quality significantly predicted doctor–patient trust via patient-perceived satisfaction (*P* = 0.050, 95% CI (0.014, 0.125)), and the ratio of the indirect effect to total effect was 11.5%. The total indirect effect of doctor–patient communication on doctor–patient trust was 0.342 (95% CI (0.191, 0.475)), which accounted for 62.41% of the total effect. Specifically, doctor–patient communication positively predicted doctor–patient trust via medical service quality (*P* = 0.004, 95% CI (0.096, 0.356)), with an estimated indirect effect of 0.190; doctor–patient communication positively predicted doctor–patient trust via service satisfaction (*P* = 0.039, 95% CI (0.034, 0.293)), with an estimated indirect effect of 0.128; and doctor–patient communication also significantly predicted doctor–patient trust via the sequential mediation variables of service quality and patient-perceived service satisfaction (*P* = 0.046, 95% CI (0.007, 0.058)), whose estimated multiple indirect effect was only 0.024. Taken together, the hypotheses in our study were all supported (Table 5).

**Table 5:** Total and indirect effects of doctor–patient communication and service quality on doctor–patient trust.

Description	Point estimate	Product of coefficients			Bootstrap 1000 times 95% CI (bias-corrected)	
		S.E	Est./S.E	*P-value*	Lower	Upper
**Effects from SQ to DPT**						
SQ → SS → DPT	0.053	0.027	1.963	0.050	0.014	0.125
Total effect	0.462	0.091	5.06	< 0.001	0.303	0.656
**Effects from DPC to DPT**						
DPC → SQ → DPT	0.190	0.065	2.91	0.004	0.096	0.356
DPC → SS → DPT	0.128	0.062	2.065	0.039	0.034	0.293
DPC → SQ → SS → DPT	0.024	0.012	1.997	0.046	0.007	0.058
Total indirect effect	0.342	0.071	4.806	< 0.001	0.191	0.475
Total effect	0.548	0.041	13.349	< 0.001	0.467	0.628

*DPC* doctor–patient communication, *SQ* service quality, *SS* service satisfaction, *DPT* doctor–patient trust, *S.E.* standard error, *CI* confidence interval, bootstrap = 1000.

## Discussion

In the process of medical service delivery, patients are on the weaker side than doctors because patients have little medical knowledge (e.g., prevention and treatment) and possess few ownership or use-rights of medical resources, which leads to the problem of trust when they must entrust their lives and health to doctors. Although doctor–patient trust is not optimistic in China, few studies have examined the effect mechanism of trust and how to establish the patient trust in the doctor. This present study explored the effects of doctor–patient communication, medical service quality, and service satisfaction on patient trust in medical staff using the SEM method.

None of the socio-demographic characteristics, except for the medical institution type, exhibited an association with doctor–patient trust. The highest level of patient trust in medical institutions was observed for the county hospital, but the lowest level was observed for tertiary specialized medical institutions, based on the results. The balance between medical service costs and the effectiveness of disease treatments are importantly related to doctor–patient trust^14^. Patients should show more trust in the county hospital, because the county hospital potentially provides better medical services than TB dispensaries and treatment costs less at the county hospital than at the tertiary hospital. Hence, the TB dispensaries should strengthen the improvement of medical technology, and the tertiary hospital should consider lower prices for medical services to improve doctor–patient trust. Additionally, the factor analysis showed that the measurements of the constructs displayed good reliability and validity, which provided a possible solution for the measurement of service quality and service satisfaction in future studies. The SEM exhibited good fit indexes, suggesting that our conceptual model was reasonable and had certain guiding value in rebuilding doctor–patient trust in practice.

In the present study, doctor–patient communication showed a significant, direct effect on doctor–patient trust (*P* = 0.016), with the highest total effect. Doctor–patient communication could be considered as the beginning of medical service delivery, which shapes the first impressions of both the doctor and patient. Additionally, effective doctor–patient communication helps to eliminate the psychological barriers and differences between doctors and patients, which all are beneficial to encouraging doctor–patient trust^50,51^. More importantly, medical service quality exerted the greatest direct effect on doctor–patient trust (*b* = 0.409, *P* < 0.001) in our study. Service quality is the core of any service industry and deserves the most attention, particularly in healthcare^52,53^. An improvement in medical service quality, namely, a safe and effective medical service, would increase the trust of patients in doctors and hospitals^52^. Moreover, consistent with the previous study, patient-perceived service satisfaction was another factor that predicted doctor–patient trust, but some studies have implied that patients were not very satisfied with the medical services in China^19,54,55^. Therefore, an urgent need is to improve patient satisfaction with medical services. Doctor–patient communication and medical service quality exerted significant positive effects on service satisfaction, providing solid evidence for methods to improve patient satisfaction in medical practice.

Furthermore, this study clarified the mediating relationship between doctor–patient communication, medical service quality, service satisfaction, and the dependent variable doctor–patient trust. First, sufficient doctor–patient communication positively influenced doctor–patient trust mediated by medical service quality or service satisfaction. According to the traditional trust theory, the interaction or communication between individuals is the starting point for the formation of all social structures, particularly interpersonal trust^56,57^. Communication between the doctor and patient is the prelude to medical delivery behaviour, in which the doctor and patient exchange information about the health check-up, diagnosis, treatment, prognosis, and other important information. Effective communication may improve the patient’s experience and perception, namely, patient-perceived service quality and satisfaction, of the medical service, which is beneficial to build the trust of patients towards doctors. Additionally, the medical service quality and service satisfaction are considered sequential mediators from doctor–patient communication to doctor–patient trust, a relationship that has not been confirmed in previous studies. After careful consideration, this finding is logical, because effective doctor–patient communication enables medical staff to better understand the patient’s health service needs, to provide better services, and thus in turn to improve patient satisfaction with medical services, which is conducive to the establishment of doctor–patient trust^4,58^.

Based on these findings, this study provided some implications for practice. Rebuilding of doctor–patient trust may be achieved by improving doctor–patient communication, medical service quality, and service satisfaction in the process of medical service delivery. First, doctor–patient communication skills must be strengthened, particularly the communication skills of doctors, which are still not very good in China^4,59^. An improvement in doctor–patient communication is conducive to the improvement of medical service quality and patient satisfaction, which are all beneficial to establishing patient trust in medical staff. The main method of doctor–patient communication in China is face-to-face communication, but the previous study reported similar for between screen-to-screen and face-to-face doctor–patient communication^60^; additionally, researchers have documented that a “Photo Stories” presentation contributes to doctor–patient communication^61^, which all provide evidence of future communication methods in China. Importantly, Chinese medical staff must develop their communication skills, such as listening to patients, engaging them in shared decision making about health, and increasing awareness of performance-related feedback in medical practice^62–64^. Certainly, patients should also seriously adhere to individual literacy in health training, and with the participation of their families, they should alleviate their discomfort and vulnerability and feel open to express themselves^65,66^.

Moreover, the service quality, which is the foundation of the medical industry, should be improved in reality, which is not only reflected in the professional skills of medical staff but also reflected in their attitudes and basic behaviours in medical service delivery. Hospitals should establish a systematic medical training and learning system to ensure that medical personnel are able to constantly improve their professional talents and cultivate a sense of responsibility, which may decrease the occurrence of lower service quality, adverse events, and medical errors^52,67^. Additionally, the medical staff should be required to establish the “patient-centred” service concept, which embodies the respect and humanistic healthcare in the whole medical service process^67^. Doctors' improper medical practices must also be monitored to provide patients with necessary medical services and products^68^. Crucially, service satisfaction plays a key role in building doctor–patient trust. Medical service satisfaction is always a concern in China, but it does not appear to have been substantially improved, even with the implementation of the medical reforms in 2009^69^. Therefore, service satisfaction should be viewed as a feedback index of the quality of medical service in future practice; on the other hand, service satisfaction should also be continuously measured and improved, which may contribute to the establishment of doctor–patient trust.

Nevertheless, several limitations need to be addressed in future studies. First, the sample analysed in this study was limited to participants from Dalian. This sample limits our ability to generalize findings to other regional groups. The problem of trust towards a doctor is not limited to patients with TB, and patients with other diseases also experience the same problem. Further studies should be extended to more diverse patients to test the adaptability of our research model. Second, the data were collected only through self-report measures, which might affect the results of this study. Patients with preferential treatment from the doctor may be more likely to provide positive feedback, which may account for reporting bias. Future studies could combine participants’ self-reported measures and content analyses by interviewing them to obtain their opinions and feedback from others. Finally, our findings were based on cross-sectional data, which limit our ability to draw a practical causal conclusion. Although the tested model provides one possible combination of the relationships, receiving more positive feedback might plausibly predict doctor–patient trust. Therefore, longitudinal studies are needed to examine causal or bi-directional relationships between these variables.

## Conclusions

This study empirically explored the effects of doctor–patient communication, medical service quality, and service satisfaction on doctor–patient trust in China, with 564 TB patients participating in the study. The results of the linear regression analysis identified doctor–patient communication, medical service quality and service satisfaction as direct predictors of doctor–patient trust. Using the SEM method, we found that (1) doctor–patient communication, medical service quality and service satisfaction directly benefited building doctor–patient trust; (2) service quality mediated the relationship between doctor–patient communication and doctor–patient trust; (3) medical service satisfaction mediated the relationship between doctor–patient communication and doctor–patient trust; (4) medical service satisfaction mediated the relationship between medical service quality and doctor–patient trust; and (5) medical service quality and service satisfaction were the sequential mediators from doctor–patient communication to doctor–patient trust. The results have completely supported the research hypotheses we proposed, and doctor–patient communication, medical service quality and service satisfaction positively predicted doctor–patient trust through a direct or indirect path, and this prediction was very good (*R-square* = 0.495). These findings not only provide theoretical support for the effect mechanism of patients' trust in medical staff but also a valid guide for rebuilding doctor–patient trust in practice.
